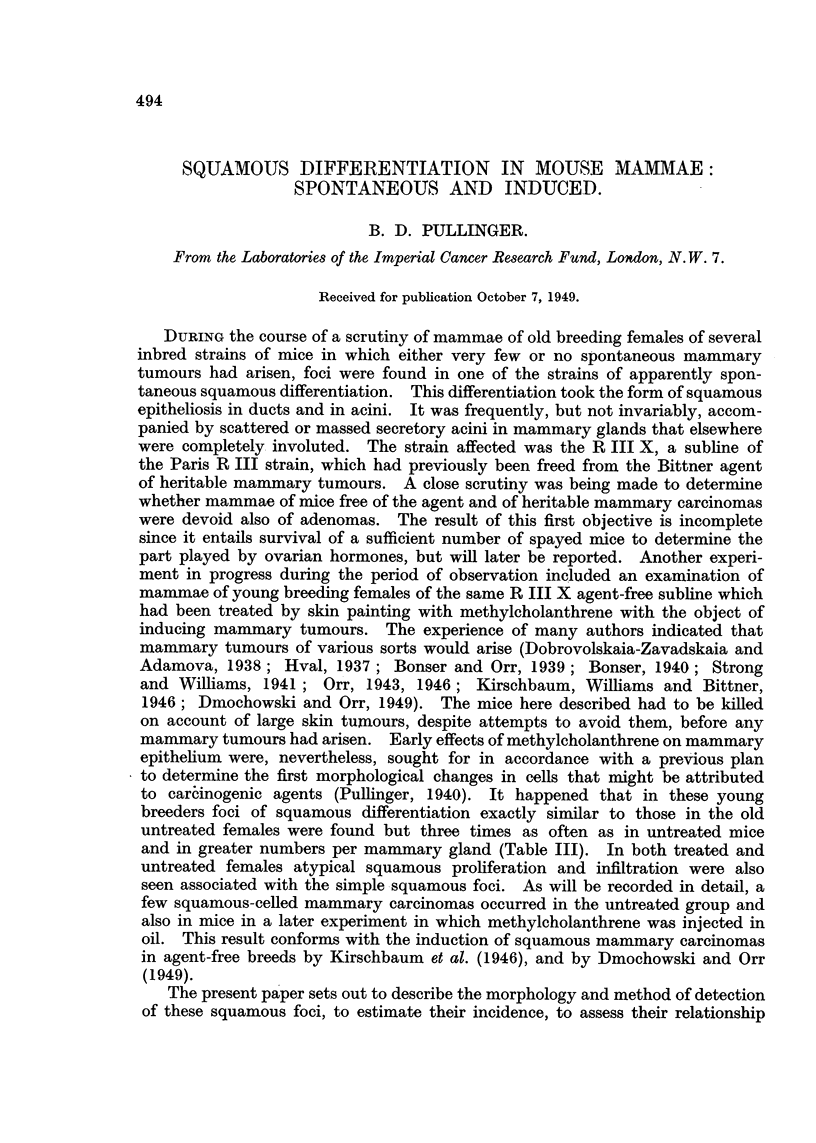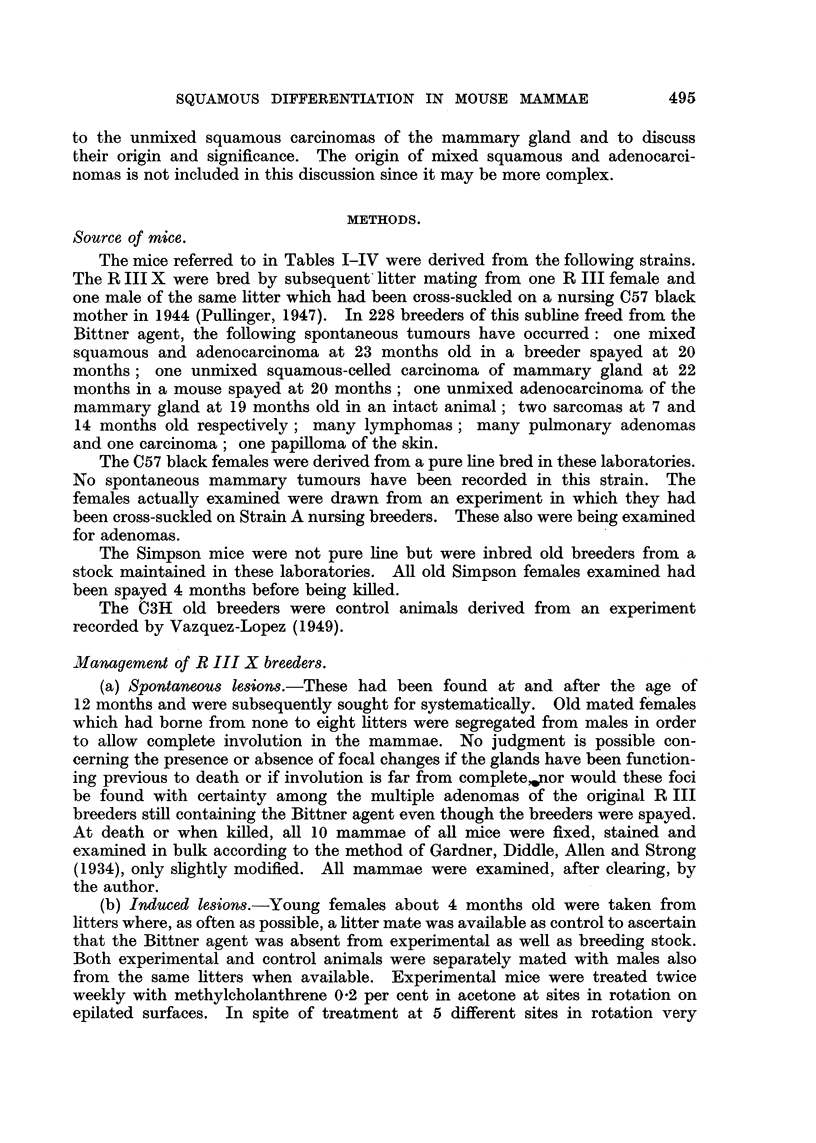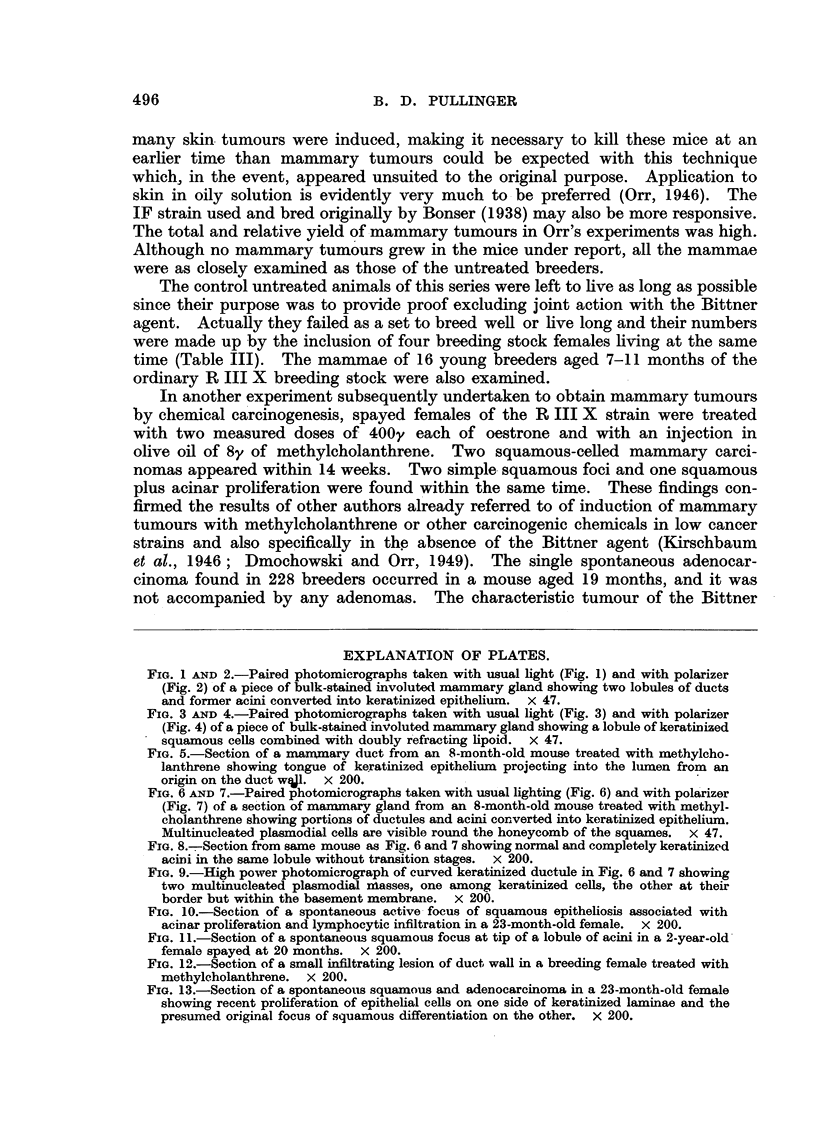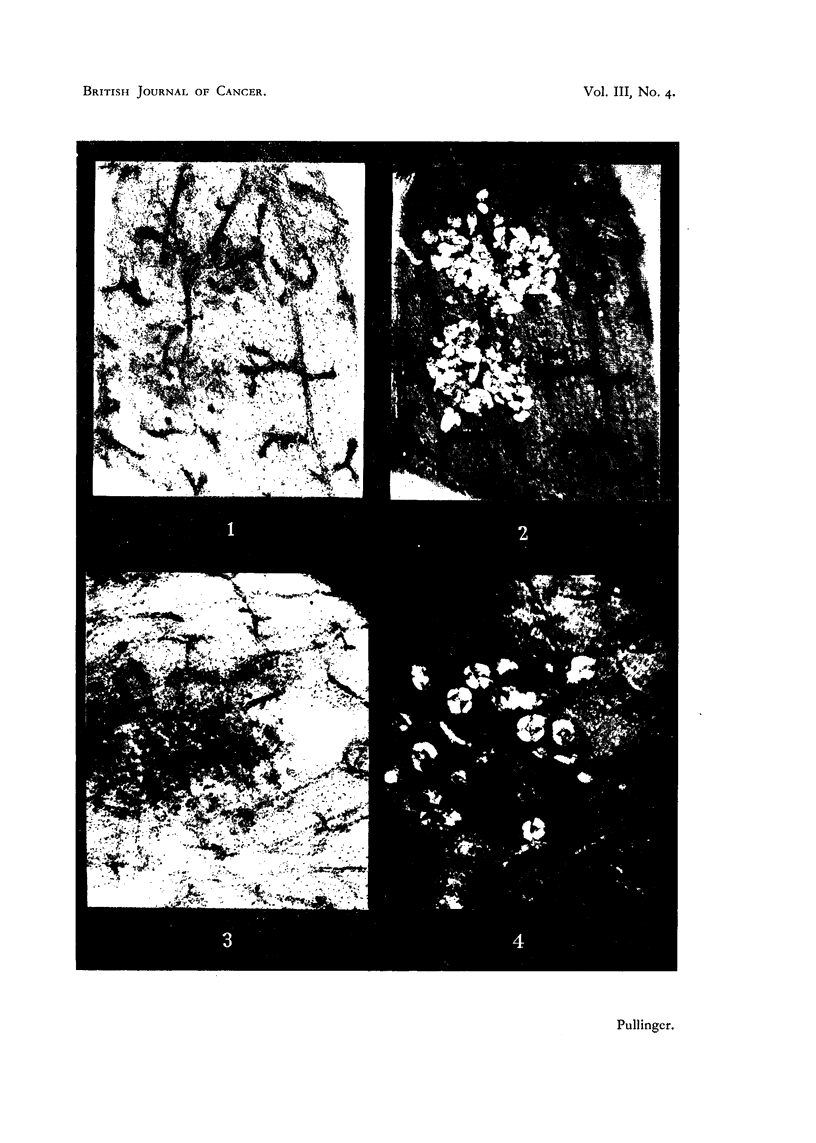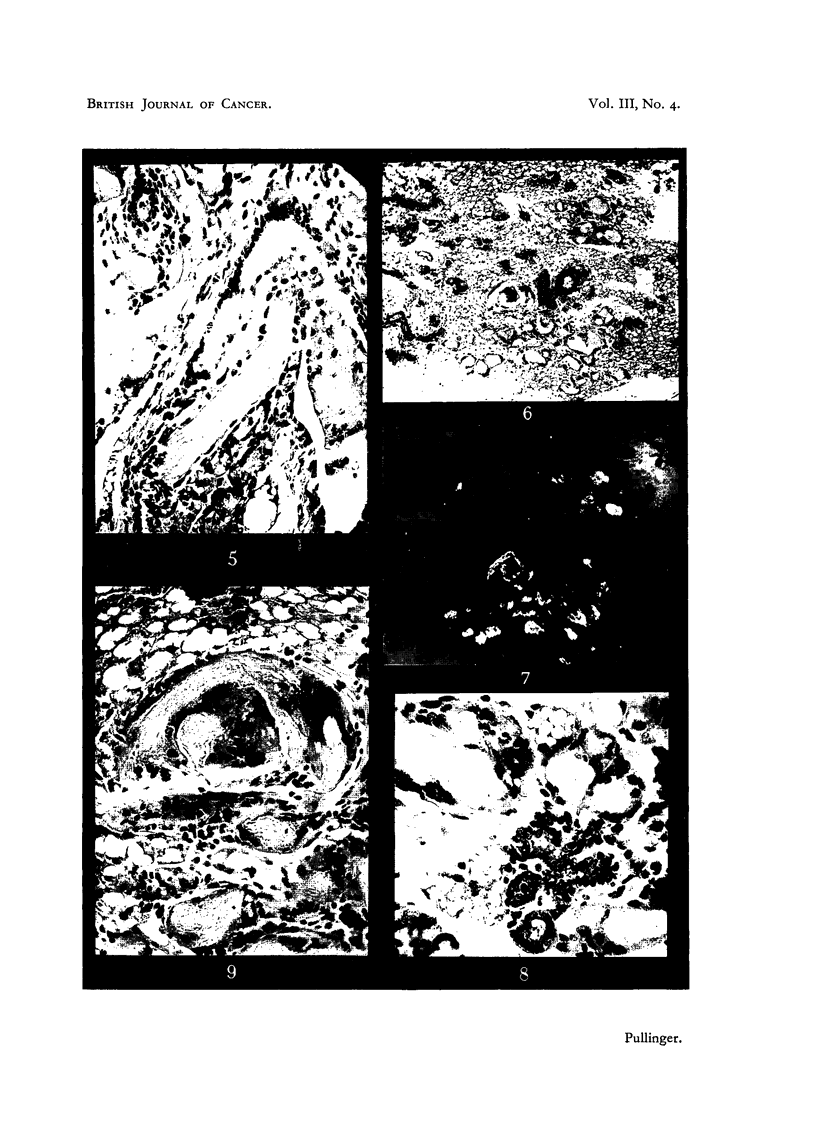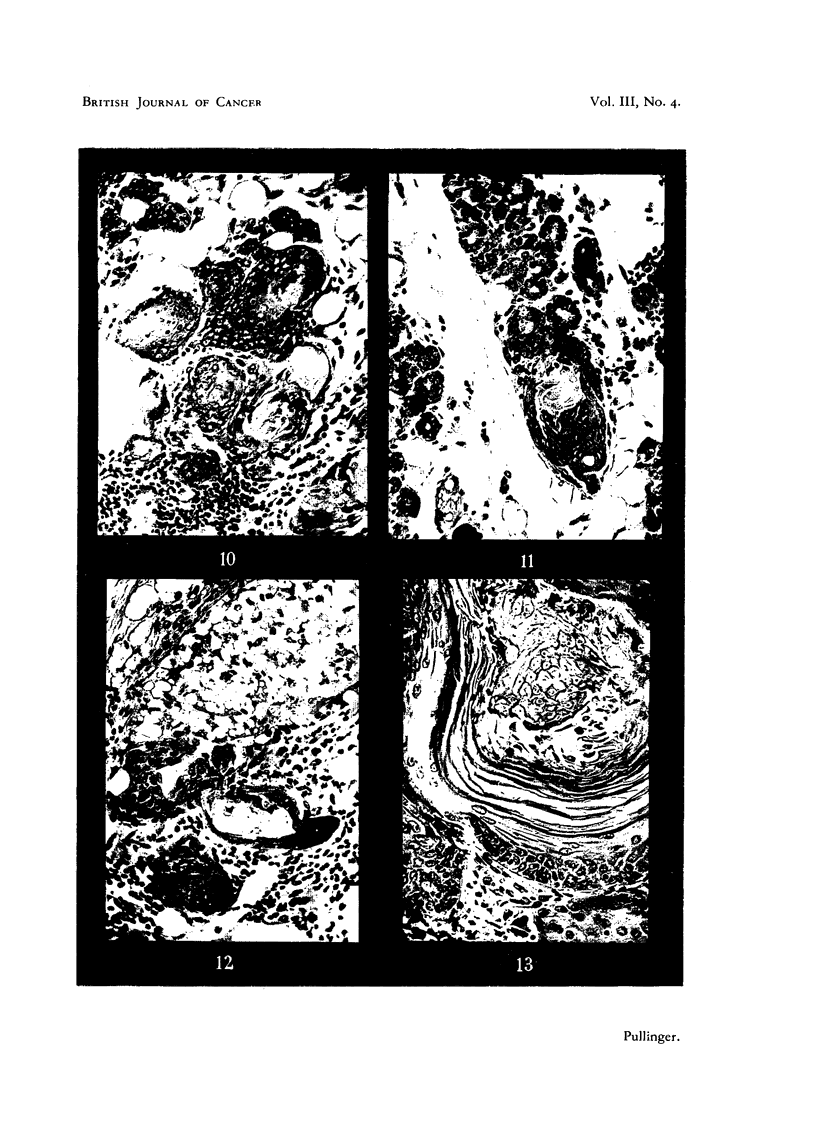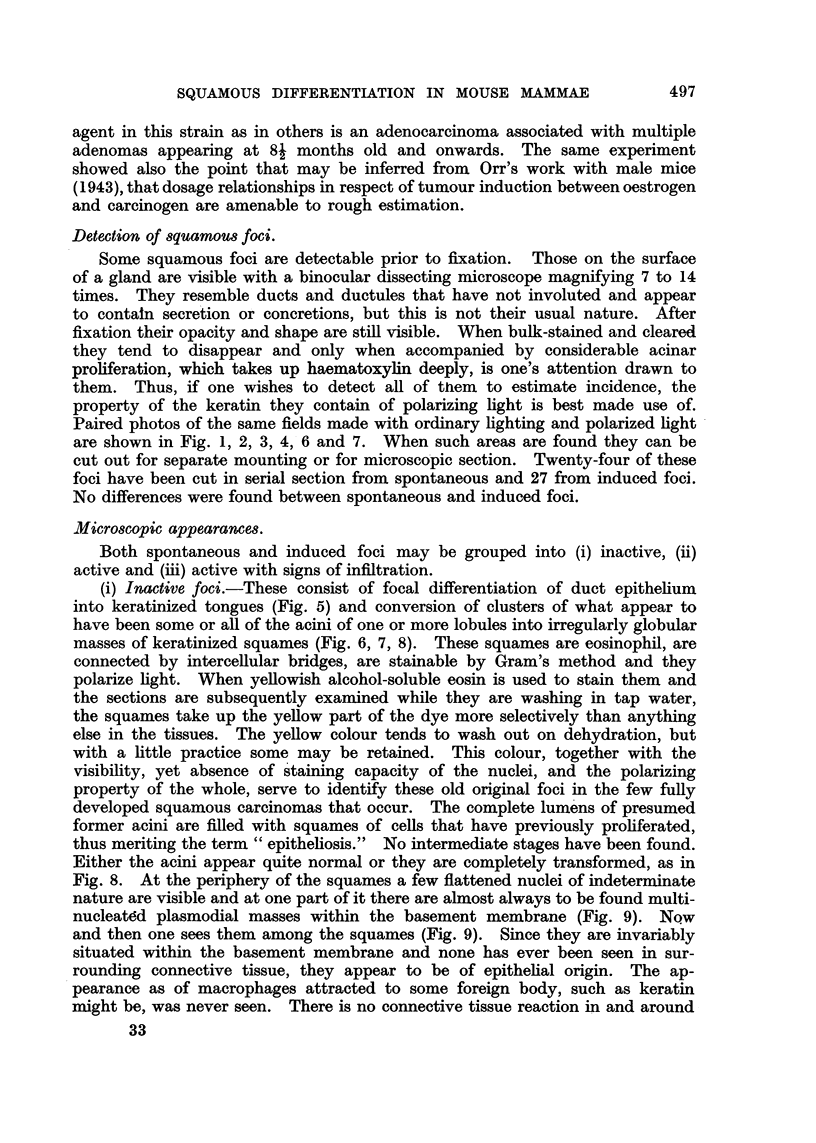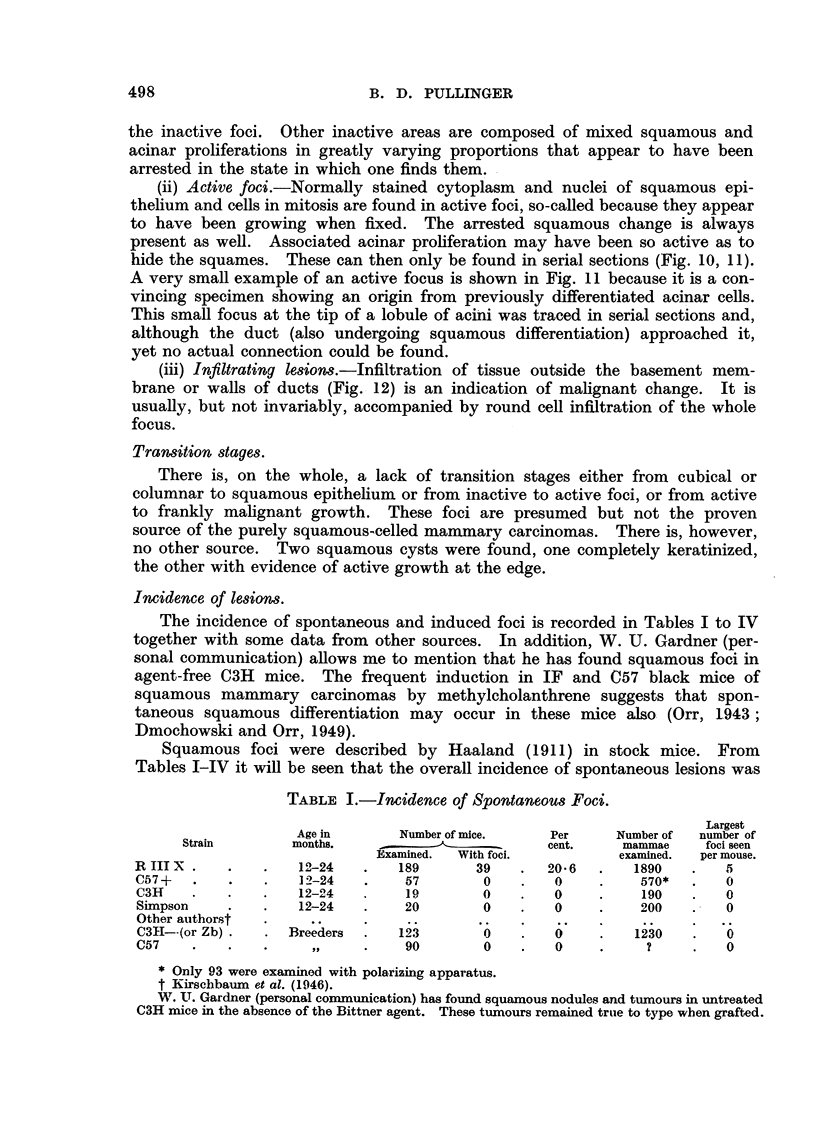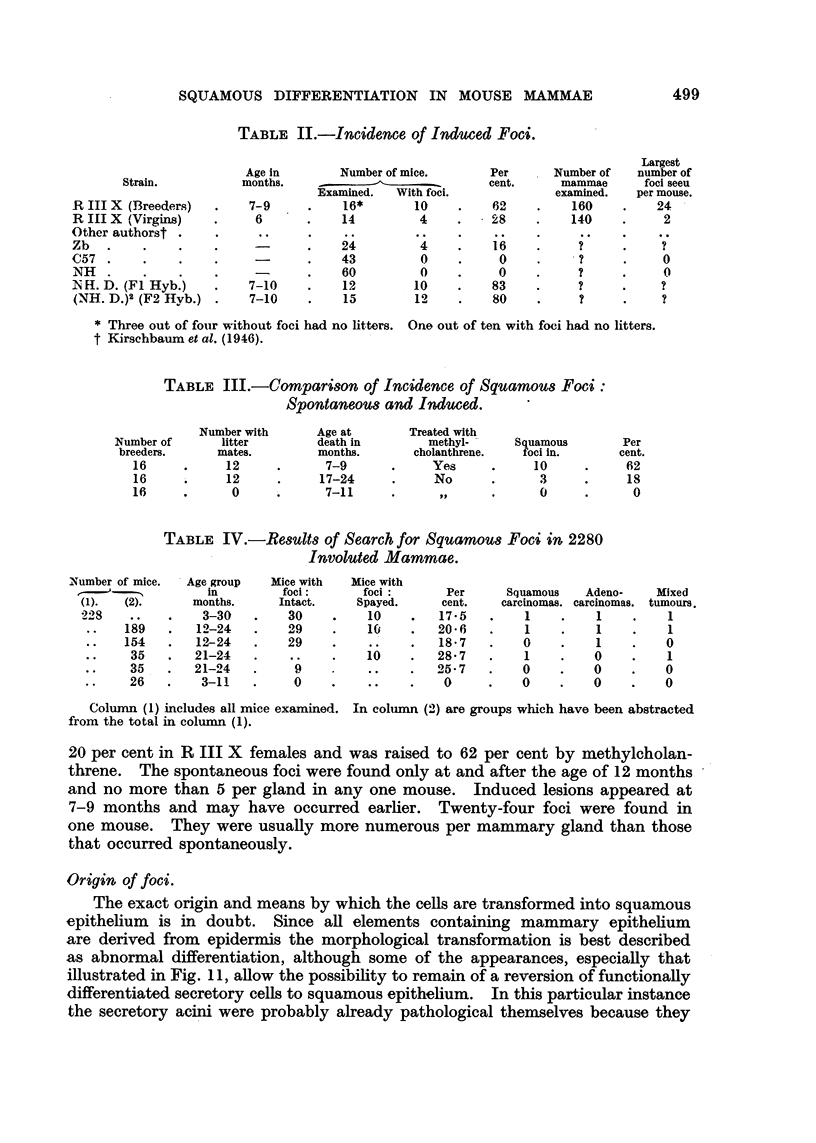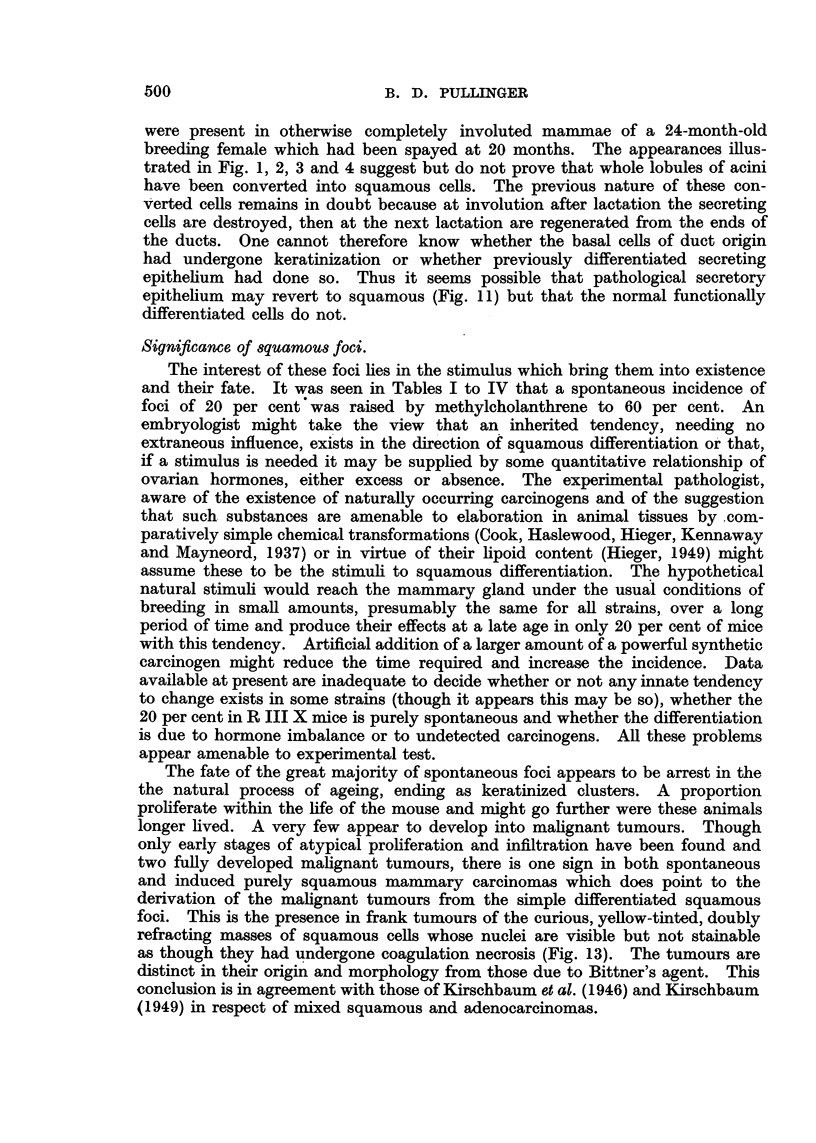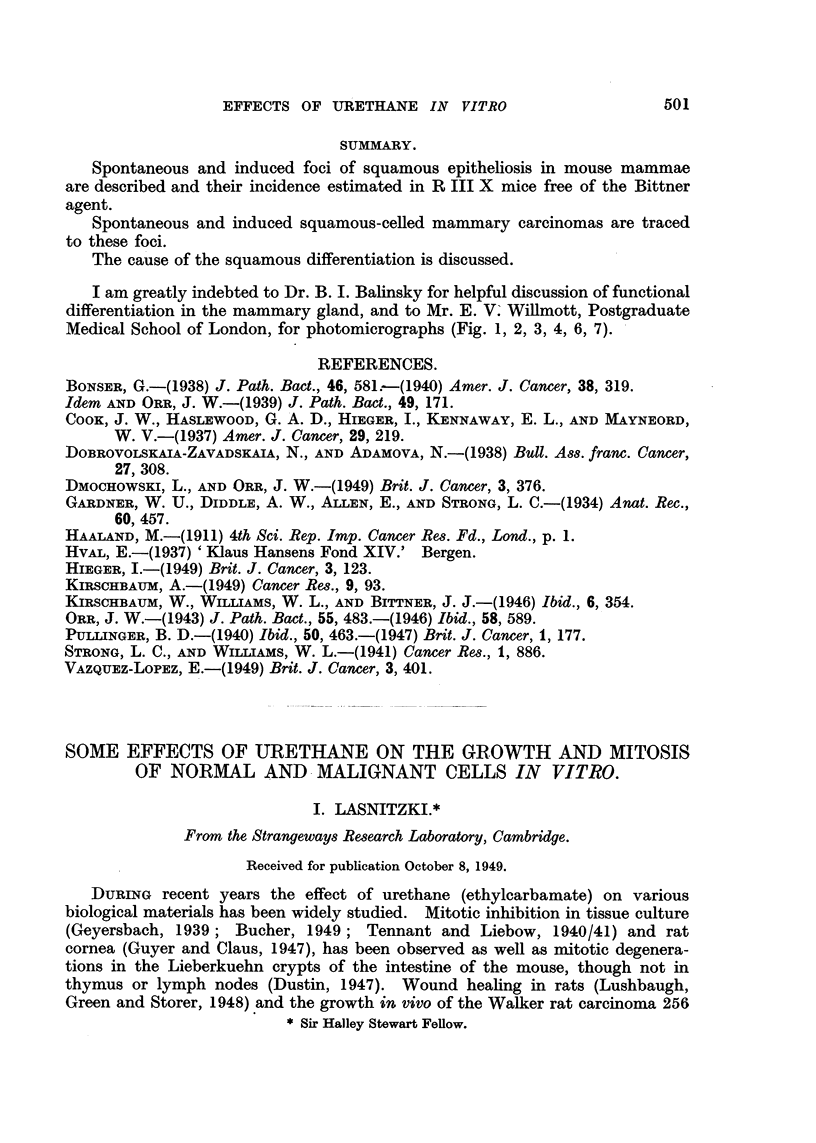# Squamous Differentiation in Mouse Mammae: Spontaneous and Induced

**DOI:** 10.1038/bjc.1949.52

**Published:** 1949-12

**Authors:** B. D. Pullinger

## Abstract

**Images:**


### SQUAMOUS DIFFERENTIATION IN MOUSE MAMMAE: SPONTANEOUS AND INDUCED.

B. D. PULLINGER.

*From the Laboratories of the Imperial Cancer Research Fund, London, N.W. 7.*

Received for publication October 7, 1949.

DURING the course of a scrutiny of mammae of old breeding females of several inbred strains of mice in which either very few or no spontaneous mammary tumours had arisen, foci were found in one of the strains of apparently spontaneous squamous differentiation. This differentiation took the form of squamous epitheliosis in ducts and in acini. It was frequently, but not invariably, accompanied by scattered or massed secretory acini in mammary glands that elsewhere were completely involuted. The strain affected was the R III X, a subline of the Paris R III strain, which had previously been freed from the Bittner agent of heritable mammary tumours. A close scrutiny was being made to determine whether mammae of mice free of the agent and of heritable mammary carcinomas were devoid also of adenomas. The result of this first objective is incomplete since it entails survival of a sufficient number of spayed mice to determine the part played by ovarian hormones, but will later be reported. Another experiment in progress during the period of observation included an examination of mammae of young breeding females of the same R III X agent-free subline which had been treated by skin painting with methylcholanthrene with the object of inducing mammary tumours. The experience of many authors indicated that mammary tumours of various sorts would arise (Dobrovolskaia-Zavadskaia and Adamova, 1938; Hval, 1937; Bonser and Orr, 1939; Bonser, 1940; Strong and Williams, 1941; Orr, 1943, 1946; Kirschbaum, Williams and Bittner, 1946; Dmochowski and Orr, 1949). The mice here described had to be killed on account of large skin tumours, despite attempts to avoid them, before any mammary tumours had arisen. Early effects of methylcholanthrene on mammary epithelium were, nevertheless, sought for in accordance with a previous plan to determine the first morphological changes in cells that might be attributed to carcinogenic agents (Pullinger, 1940). It happened that in these young breeders foci of squamous differentiation exactly similar to those in the old untreated females were found but three times as often as in untreated mice and in greater numbers per mammary gland (Table III). In both treated and untreated females atypical squamous proliferation and infiltration were also seen associated with the simple squamous foci. As will be recorded in detail, a few squamous-celled mammary carcinomas occurred in the untreated group and also in mice in a later experiment in which methylcholanthrene was injected in oil. This result conforms with the induction of squamous mammary carcinomas in agent-free breeds by Kirschbaum *et al.* (1946), and by Dmochowski and Orr (1949).

The present paper sets out to describe the morphology and method of detection of these squamous foci, to estimate their incidence, to assess their relationship to the unmixed squamous carcinomas of the mammary gland and to discuss their origin and significance. The origin of mixed squamous and adenocarcinomas is not included in this discussion since it may be more complex.

### METHODS.

*Source of mice.*

The mice referred to in Tables I–IV were derived from the following strains. The R III X were bred by subsequent litter mating from one R III female and one male of the same litter which had been cross-suckled on a nursing C57 black mother in 1944 (Pullinger, 1947). In 228 breeders of this subline freed from the Bittner agent, the following spontaneous tumours have occurred : one mixed squamous and adenocarcinoma at 23 months old in a breeder spayed at 20 months ; one unmixed squamous-celled carcinoma of mammary gland at 22 months in a mouse spayed at 20 months ; one unmixed adenocarcinoma of the mammary gland at 19 months old in an intact animal ; two sarcomas at 7 and 14 months old respectively ; many lymphomas ; many pulmonary adenomas and one carcinoma ; one papilloma of the skin.

The C57 black females were derived from a pure line bred in these laboratories. No spontaneous mammary tumours have been recorded in this strain. The females actually examined were drawn from an experiment in which they had been cross-suckled on Strain A nursing breeders. These also were being examined for adenomas.

The Simpson mice were not pure line but were inbred old breeders from a stock maintained in these laboratories. All old Simpson females examined had been spayed 4 months before being killed.

The C3H old breeders were control animals derived from an experiment recorded by Vazquez-Lopez (1949).

*Management of R III X breeders.*

(a) *Spontaneous lesions.*—These had been found at and after the age of 12 months and were subsequently sought for systematically. Old mated females which had borne from none to eight litters were segregated from males in order to allow complete involution in the mammae. No judgment is possible concerning the presence or absence of focal changes if the glands have been functioning previous to death or if involution is far from complete, nor would these foci be found with certainty among the multiple adenomas of the original R III breeders still containing the Bittner agent even though the breeders were spayed. At death or when killed, all 10 mammae of all mice were fixed, stained and examined in bulk according to the method of Gardner, Diddle, Allen and Strong (1934), only slightly modified. All mammae were examined, after clearing, by the author.

(b) *Induced lesions.*—Young females about 4 months old were taken from litters where, as often as possible, a litter mate was available as control to ascertain that the Bittner agent was absent from experimental as well as breeding stock. Both experimental and control animals were separately mated with males also from the same litters when available. Experimental mice were treated twice weekly with methylcholanthrene 0·2 per cent in acetone at sites in rotation on epilated surfaces. In spite of treatment at 5 different sites in rotation very many skin tumours were induced, making it necessary to kill these mice at an earlier time than mammary tumours could be expected with this technique which, in the event, appeared unsuited to the original purpose. Application to skin in oily solution is evidently very much to be preferred (Orr, 1946). The IF strain used and bred originally by Bonser (1938) may also be more responsive. The total and relative yield of mammary tumours in Orr's experiments was high. Although no mammary tumours grew in the mice under report, all the mammae were as closely examined as those of the untreated breeders.

The control untreated animals of this series were left to live as long as possible since their purpose was to provide proof excluding joint action with the Bittner agent. Actually they failed as a set to breed well or live long and their numbers were made up by the inclusion of four breeding stock females living at the same time (Table III). The mammae of 16 young breeders aged 7–11 months of the ordinary R III X breeding stock were also examined.

In another experiment subsequently undertaken to obtain mammary tumours by chemical carcinogenesis, spayed females of the R III X strain were treated with two measured doses of 400$\gamma$ each of oestrone and with an injection in olive oil of 8$\gamma$ of methylcholanthrene. Two squamous-celled mammary carcinomas appeared within 14 weeks. Two simple squamous foci and one squamous plus acinar proliferation were found within the same time. These findings confirmed the results of other authors already referred to of induction of mammary tumours with methylcholanthrene or other carcinogenic chemicals in low cancer strains and also specifically in the absence of the Bittner agent (Kirschbaum *et al.*, 1946; Dmochowski and Orr, 1949). The single spontaneous adenocarcinoma found in 228 breeders occurred in a mouse aged 19 months, and it was not accompanied by any adenomas. The characteristic tumour of the Bittner

---

EXPLANATION OF PLATES.

FIG. 1 AND 2.—Paired photomicrographs taken with usual light (Fig. 1) and with polarizer (Fig. 2) of a piece of bulk-stained involuted mammary gland showing two lobules of ducts and former acini converted into keratinized epithelium. × 47.

FIG. 3 AND 4.—Paired photomicrographs taken with usual light (Fig. 3) and with polarizer (Fig. 4) of a piece of bulk-stained involuted mammary gland showing a lobule of keratinized squamous cells combined with doubly refracting lipoid. × 47.

FIG. 5.—Section of a mammary duct from an 8-month-old mouse treated with methylcholanthrene showing tongue of keratinized epithelium projecting into the lumen from an origin on the duct wall. × 200.

FIG. 6 AND 7.—Paired photomicrographs taken with usual lighting (Fig. 6) and with polarizer (Fig. 7) of a section of mammary gland from an 8-month-old mouse treated with methylcholanthrene showing portions of ductules and acini converted into keratinized epithelium. Multinucleated plasmodial cells are visible round the honeycomb of the squames. × 47.

FIG. 8.—Section from same mouse as Fig. 6 and 7 showing normal and completely keratinized acini in the same lobule without transition stages. × 200.

FIG. 9.—High power photomicrograph of curved keratinized ductule in Fig. 6 and 7 showing two multinucleated plasmodial masses, one among keratinized cells, the other at their border but within the basement membrane. × 200.

FIG. 10.—Section of a spontaneous active focus of squamous epitheliosis associated with acinar proliferation and lymphocytic infiltration in a 23-month-old female. × 200.

FIG. 11.—Section of a spontaneous squamous focus at tip of a lobule of acini in a 2-year-old female spayed at 20 months. × 200.

FIG. 12.—Section of a small infiltrating lesion of duct wall in a breeding female treated with methylcholanthrene. × 200.

FIG. 13.—Section of a spontaneous squamous and adenocarcinoma in a 23-month-old female showing recent proliferation of epithelial cells on one side of keratinized laminae and the presumed original focus of squamous differentiation on the other. × 200.

1

2

3

4

Pullinger.

5

6

7

9

8

10

11

12

13

Pullinger.

agent in this strain as in others is an adenocarcinoma associated with multiple adenomas appearing at 8½ months old and onwards. The same experiment showed also the point that may be inferred from Orr's work with male mice (1943), that dosage relationships in respect of tumour induction between oestrogen and carcinogen are amenable to rough estimation.

*Detection of squamous foci.*

Some squamous foci are detectable prior to fixation. Those on the surface of a gland are visible with a binocular dissecting microscope magnifying 7 to 14 times. They resemble ducts and ductules that have not involuted and appear to contain secretion or concretions, but this is not their usual nature. After fixation their opacity and shape are still visible. When bulk-stained and cleared they tend to disappear and only when accompanied by considerable acinar proliferation, which takes up haematoxylin deeply, is one's attention drawn to them. Thus, if one wishes to detect all of them to estimate incidence, the property of the keratin they contain of polarizing light is best made use of. Paired photos of the same fields made with ordinary lighting and polarized light are shown in Fig. 1, 2, 3, 4, 6 and 7. When such areas are found they can be cut out for separate mounting or for microscopic section. Twenty-four of these foci have been cut in serial section from spontaneous and 27 from induced foci. No differences were found between spontaneous and induced foci.

*Microscopic appearances.*

Both spontaneous and induced foci may be grouped into (i) inactive, (ii) active and (iii) active with signs of infiltration.

(i) *Inactive foci.*—These consist of focal differentiation of duct epithelium into keratinized tongues (Fig. 5) and conversion of clusters of what appear to have been some or all of the acini of one or more lobules into irregularly globular masses of keratinized squames (Fig. 6, 7, 8). These squames are eosinophil, are connected by intercellular bridges, are stainable by Gram's method and they polarize light. When yellowish alcohol-soluble eosin is used to stain them and the sections are subsequently examined while they are washing in tap water, the squames take up the yellow part of the dye more selectively than anything else in the tissues. The yellow colour tends to wash out on dehydration, but with a little practice some may be retained. This colour, together with the visibility, yet absence of staining capacity of the nuclei, and the polarizing property of the whole, serve to identify these old original foci in the few fully developed squamous carcinomas that occur. The complete lumens of presumed former acini are filled with squames of cells that have previously proliferated, thus meriting the term "epitheliosis." No intermediate stages have been found. Either the acini appear quite normal or they are completely transformed, as in Fig. 8. At the periphery of the squames a few flattened nuclei of indeterminate nature are visible and at one part of it there are almost always to be found multinucleated plasmodial masses within the basement membrane (Fig. 9). Now and then one sees them among the squames (Fig. 9). Since they are invariably situated within the basement membrane and none has ever been seen in surrounding connective tissue, they appear to be of epithelial origin. The appearance as of macrophages attracted to some foreign body, such as keratin might be, was never seen. There is no connective tissue reaction in and around the inactive foci. Other inactive areas are composed of mixed squamous and acinar proliferations in greatly varying proportions that appear to have been arrested in the state in which one finds them.

(ii) *Active foci.*—Normally stained cytoplasm and nuclei of squamous epithelium and cells in mitosis are found in active foci, so-called because they appear to have been growing when fixed. The arrested squamous change is always present as well. Associated acinar proliferation may have been so active as to hide the squames. These can then only be found in serial sections (Fig. 10, 11). A very small example of an active focus is shown in Fig. 11 because it is a convincing specimen showing an origin from previously differentiated acinar cells. This small focus at the tip of a lobule of acini was traced in serial sections and, although the duct (also undergoing squamous differentiation) approached it, yet no actual connection could be found.

(iii) *Infiltrating lesions.*—Infiltration of tissue outside the basement membrane or walls of ducts (Fig. 12) is an indication of malignant change. It is usually, but not invariably, accompanied by round cell infiltration of the whole focus.

*Transition stages.*

There is, on the whole, a lack of transition stages either from cubical or columnar to squamous epithelium or from inactive to active foci, or from active to frankly malignant growth. These foci are presumed but not the proven source of the purely squamous-celled mammary carcinomas. There is, however, no other source. Two squamous cysts were found, one completely keratinized, the other with evidence of active growth at the edge.

*Incidence of lesions.*

The incidence of spontaneous and induced foci is recorded in Tables I to IV together with some data from other sources. In addition, W. U. Gardner (personal communication) allows me to mention that he has found squamous foci in agent-free C3H mice. The frequent induction in IF and C57 black mice of squamous mammary carcinomas by methylcholanthrene suggests that spontaneous squamous differentiation may occur in these mice also (Orr, 1943 ; Dmochowski and Orr, 1949).

Squamous foci were described by Haaland (1911) in stock mice. From Tables I–IV it will be seen that the overall incidence of spontaneous lesions was

TABLE I.—*Incidence of Spontaneous Foci.*

| Strain | Age in months. | Number of mice. Examined. | Number of mice. With foci. | Per cent. | Number of mammae examined. | Largest number of foci seen per mouse. |
|---|---|---|---|---|---|---|
| R III X | 12–24 | 189 | 39 | 20·6 | 1890 | 5 |
| C57+ | 12–24 | 57 | 0 | 0 | 570* | 0 |
| C3H | 12–24 | 19 | 0 | 0 | 190 | 0 |
| Simpson | 12–24 | 20 | 0 | 0 | 200 | 0 |
| Other authors† | .. | .. | .. | .. | .. | .. |
| C3H—(or Zb) | Breeders | 123 | 0 | 0 | 1230 | 0 |
| C57 | ,, | 90 | 0 | 0 | ? | 0 |

* Only 93 were examined with polarizing apparatus.
† Kirschbaum *et al.* (1946).
W. U. Gardner (personal communication) has found squamous nodules and tumours in untreated C3H mice in the absence of the Bittner agent. These tumours remained true to type when grafted.

TABLE II.—*Incidence of Induced Foci.*

| Strain. | Age in months. | Number of mice. | | Per cent. | Number of mammae examined. | Largest number of foci seeu per mouse. |
|---|---|---|---|---|---|---|
| | | Examined. | With foci. | | | |
| R III X (Breeders) | 7–9 | 16* | 10 | 62 | 160 | 24 |
| R III X (Virgins) | 6 | 14 | 4 | 28 | 140 | 2 |
| Other authors† | .. | .. | .. | .. | .. | .. |
| Zb | — | 24 | 4 | 16 | ? | ? |
| C57 | — | 43 | 0 | 0 | ? | 0 |
| NH | — | 60 | 0 | 0 | ? | 0 |
| NH. D. (F1 Hyb.) | 7–10 | 12 | 10 | 83 | ? | ? |
| (NH. D.)$^2$ (F2 Hyb.) | 7–10 | 15 | 12 | 80 | ? | ? |

* Three out of four without foci had no litters. One out of ten with foci had no litters.
† Kirschbaum *et al.* (1946).

TABLE III.—*Comparison of Incidence of Squamous Foci : Spontaneous and Induced.*

| Number of breeders. | Number with litter mates. | Age at death in months. | Treated with methyl-cholanthrene. | Squamous foci in. | Per cent. |
|---|---|---|---|---|---|
| 16 | 12 | 7–9 | Yes | 10 | 62 |
| 16 | 12 | 17–24 | No | 3 | 18 |
| 16 | 0 | 7–11 | ,, | 0 | 0 |

TABLE IV.—*Results of Search for Squamous Foci in* 2280 *Involuted Mammae.*

| Number of mice. (1). | Number of mice. (2). | Age group in months. | Mice with foci : Intact. | Mice with foci : Spayed. | Per cent. | Squamous carcinomas. | Adeno-carcinomas. | Mixed tumours. |
|---|---|---|---|---|---|---|---|---|
| 228 | .. | 3–30 | 30 | 10 | 17·5 | 1 | 1 | 1 |
| .. | 189 | 12–24 | 29 | 10 | 20·6 | 1 | 1 | 1 |
| .. | 154 | 12–24 | 29 | .. | 18·7 | 0 | 1 | 0 |
| .. | 35 | 21–24 | .. | 10 | 28·7 | 1 | 0 | 1 |
| .. | 35 | 21–24 | 9 | .. | 25·7 | 0 | 0 | 0 |
| .. | 26 | 3–11 | 0 | .. | 0 | 0 | 0 | 0 |

Column (1) includes all mice examined. In column (2) are groups which have been abstracted from the total in column (1).

20 per cent in R III X females and was raised to 62 per cent by methylcholanthrene. The spontaneous foci were found only at and after the age of 12 months and no more than 5 per gland in any one mouse. Induced lesions appeared at 7–9 months and may have occurred earlier. Twenty-four foci were found in one mouse. They were usually more numerous per mammary gland than those that occurred spontaneously.

*Origin of foci.*

The exact origin and means by which the cells are transformed into squamous epithelium is in doubt. Since all elements containing mammary epithelium are derived from epidermis the morphological transformation is best described as abnormal differentiation, although some of the appearances, especially that illustrated in Fig. 11, allow the possibility to remain of a reversion of functionally differentiated secretory cells to squamous epithelium. In this particular instance the secretory acini were probably already pathological themselves because they were present in otherwise completely involuted mammae of a 24-month-old breeding female which had been spayed at 20 months. The appearances illustrated in Fig. 1, 2, 3 and 4 suggest but do not prove that whole lobules of acini have been converted into squamous cells. The previous nature of these converted cells remains in doubt because at involution after lactation the secreting cells are destroyed, then at the next lactation are regenerated from the ends of the ducts. One cannot therefore know whether the basal cells of duct origin had undergone keratinization or whether previously differentiated secreting epithelium had done so. Thus it seems possible that pathological secretory epithelium may revert to squamous (Fig. 11) but that the normal functionally differentiated cells do not.

*Significance of squamous foci.*

The interest of these foci lies in the stimulus which bring them into existence and their fate. It was seen in Tables I to IV that a spontaneous incidence of foci of 20 per cent was raised by methylcholanthrene to 60 per cent. An embryologist might take the view that an inherited tendency, needing no extraneous influence, exists in the direction of squamous differentiation or that, if a stimulus is needed it may be supplied by some quantitative relationship of ovarian hormones, either excess or absence. The experimental pathologist, aware of the existence of naturally occurring carcinogens and of the suggestion that such substances are amenable to elaboration in animal tissues by comparatively simple chemical transformations (Cook, Haslewood, Hieger, Kennaway and Mayneord, 1937) or in virtue of their lipoid content (Hieger, 1949) might assume these to be the stimuli to squamous differentiation. The hypothetical natural stimuli would reach the mammary gland under the usual conditions of breeding in small amounts, presumably the same for all strains, over a long period of time and produce their effects at a late age in only 20 per cent of mice with this tendency. Artificial addition of a larger amount of a powerful synthetic carcinogen might reduce the time required and increase the incidence. Data available at present are inadequate to decide whether or not any innate tendency to change exists in some strains (though it appears this may be so), whether the 20 per cent in R III X mice is purely spontaneous and whether the differentiation is due to hormone imbalance or to undetected carcinogens. All these problems appear amenable to experimental test.

The fate of the great majority of spontaneous foci appears to be arrest in the the natural process of ageing, ending as keratinized clusters. A proportion proliferate within the life of the mouse and might go further were these animals longer lived. A very few appear to develop into malignant tumours. Though only early stages of atypical proliferation and infiltration have been found and two fully developed malignant tumours, there is one sign in both spontaneous and induced purely squamous mammary carcinomas which does point to the derivation of the malignant tumours from the simple differentiated squamous foci. This is the presence in frank tumours of the curious, yellow-tinted, doubly refracting masses of squamous cells whose nuclei are visible but not stainable as though they had undergone coagulation necrosis (Fig. 13). The tumours are distinct in their origin and morphology from those due to Bittner's agent. This conclusion is in agreement with those of Kirschbaum *et al.* (1946) and Kirschbaum (1949) in respect of mixed squamous and adenocarcinomas.

SUMMARY.

Spontaneous and induced foci of squamous epitheliosis in mouse mammae are described and their incidence estimated in R III X mice free of the Bittner agent.

Spontaneous and induced squamous-celled mammary carcinomas are traced to these foci.

The cause of the squamous differentiation is discussed.

I am greatly indebted to Dr. B. I. Balinsky for helpful discussion of functional differentiation in the mammary gland, and to Mr. E. V. Willmott, Postgraduate Medical School of London, for photomicrographs (Fig. 1, 2, 3, 4, 6, 7).

REFERENCES.

BONSER, G.—(1938) *J. Path. Bact.*, **46**, 581.—(1940) *Amer. J. Cancer*, **38**, 319.
*Idem* AND ORR, J. W.—(1939) *J. Path. Bact.*, **49**, 171.
COOK, J. W., HASLEWOOD, G. A. D., HIEGER, I., KENNAWAY, E. L., AND MAYNEORD, W. V.—(1937) *Amer. J. Cancer*, **29**, 219.
DOBROVOLSKAIA-ZAVADSKAIA, N., AND ADAMOVA, N.—(1938) *Bull. Ass. franc. Cancer*, **27**, 308.
DMOCHOWSKI, L., AND ORR, J. W.—(1949) *Brit. J. Cancer*, **3**, 376.
GARDNER, W. U., DIDDLE, A. W., ALLEN, E., AND STRONG, L. C.—(1934) *Anat. Rec.*, **60**, 457.
HAALAND, M.—(1911) *4th Sci. Rep. Imp. Cancer Res. Fd., Lond.*, p. 1.
HVAL, E.—(1937) 'Klaus Hansens Fond XIV.' Bergen.
HIEGER, I.—(1949) *Brit. J. Cancer*, **3**, 123.
KIRSCHBAUM, A.—(1949) *Cancer Res.*, **9**, 93.
KIRSCHBAUM, W., WILLIAMS, W. L., AND BITTNER, J. J.—(1946) *Ibid.*, **6**, 354.
ORR, J. W.—(1943) *J. Path. Bact.*, **55**, 483.—(1946) *Ibid.*, **58**, 589.
PULLINGER, B. D.—(1940) *Ibid.*, **50**, 463.—(1947) *Brit. J. Cancer*, **1**, 177.
STRONG, L. C., AND WILLIAMS, W. L.—(1941) *Cancer Res.*, **1**, 886.
VAZQUEZ-LOPEZ, E.—(1949) *Brit. J. Cancer*, **3**, 401.